# Exploring the Role of T‐Cell Metabolism in Modulating Immunotherapy Efficacy for Non‐Small Cell Lung Cancer Based on Clustering

**DOI:** 10.1002/jcla.25020

**Published:** 2025-04-17

**Authors:** Hongzhe Guo, Liangyu Zhang, Hu Tang, Peiwen Liu, Bin Hu, Yue Gong, Rui Hou, Ziheng Wu

**Affiliations:** ^1^ School of Electrical and Information Engineering Anhui University of Technology Maanshan China; ^2^ Department of Medical Oncology The General Hospital of Daqing Oil Field Daqing China; ^3^ The Second Affiliated Hospital Zhejiang University School of Medicine Hangzhou China; ^4^ School of Science Zhejiang Sci‐Tech University Hangzhou China; ^5^ Department of Thoracic Surgery, Beijing Chaoyang Hospital Capital Medical University Beijing China; ^6^ Geneis Beijing Co., Ltd. Beijing China

**Keywords:** clustering, immune metabolism, immunotherapy, non‐small cell lung cancer, T cell

## Abstract

**Background:**

Immunotherapy, especially immune checkpoint blockade (ICB) therapy, has demonstrated noteworthy advancements in the realm of non‐small cell lung cancer (NSCLC). However, the efficacy of ICB therapy is limited to a small subset of patients with NSCLC, and the underlying mechanisms remain poorly understood.

**Study Design and Discoveries:**

In this study, we conducted a comprehensive investigation of the metabolic profiles of infiltrating T cells in NSCLC tumors and revealed the metabolic heterogeneity, which associated with the prognosis of ICB therapy, in three T‐cell subtypes. After metabolic clustering, we split these metabolic clusters into two groups: Nonresponse‐associated (NR) clusters that enriched with cells from nonresponders, and response‐associated (R) clusters that not belonging to NR clusters. Then, we elucidated their metabolic differences and specific functions. Notably, we discovered *HSPA1A* was significantly downregulated in NR clusters of all three T‐cell subtypes. In addition, leveraging single‐cell T‐cell receptor sequencing data and pseudotime series analysis, we revealed the reciprocal interconversion between R and NR metabolic clusters within the same T‐cell clone. This suggests a potential metabolic reprogramming capability of T cells. Furthermore, through the analysis of intercellular communication, we identified the specific intercellular signaling in the R clusters, which might promote the activation and regulation of signal transduction pathways that affect the prognosis of ICB therapy.

**Conclusion:**

In conclusion, our study offers substantial insights into the mechanisms of relationships between T‐cell metabolisms and ICB therapy outcomes, shedding light on the mechanism of immunotherapy efficacy in patients with NSCLC. Such investigations will contribute to overcoming treatment resistance.

## Graphical abstract

We conducted a comprehensive investigation of the metabolic profiles of infiltrating T cells in NSCLC tumors and found metabolic heterogeneity in three T‐cell subtypes, which are associated with the prognosis of ICB therapy. We defined these metabolic clusters as response‐dependent (R) clusters and non‐response‐dependent (NR) clusters, and elucidated their metabolic differences and specific functions. Notably, we discovered significant downregulation of HSPA1A in the NR cluster of all three T‐cell subtypes. In addition, leveraging single‐cell T‐cell receptor sequencing (scTCR‐seq) data and pseudo‐time series analysis, we revealed the reciprocal interconversion between the two metabolic clusters within the same T‐cell clone. This suggests a potential metabolic reprogramming capability of T cells. Furthermore, through the analysis of intercellular communication, we identified the specific intercellular signaling in the R clusters, which may promote the activation and regulation of signal transduction pathways that affect the prognosis of immunotherapy.

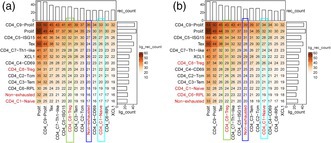

## 1 Introduction

As one of the most common cancer types, lung cancer (LC) is a devastating disease with more than 2 million new cases diagnosed all over the world every year [1, 2]. Annually, more than 1.4 million deaths are caused by LC, which is the leading cause of cancer‐related death [3]. The treatments for LC include chemotherapy, radiation therapy, targeted therapy, immunotherapy, and surgery. Chemotherapy involves drugs usage to destroy cancer cells in either alone or combined therapy [4]. Radiation therapy uses high‐energy rays, such as X‐rays or proton beam, to kill cancer cells [5]. Targeted therapy is engineered to specifically target mutations that foster the survival and proliferation of cancer cells [6]. Immunotherapy harnesses the immune system to combat cancer by activating the body's natural defences [7]. Surgery involves the removal of tumors from the body during a medical procedure [8]. Depending on the severity of LC, treatment may encompass one or more of these modalities.

Immunotherapy for LC is an evolving treatment option that is becoming increasingly popular these years [9]. There are several types of immunotherapy treatments, including monoclonal antibodies, cancer vaccines, and adoptive cell transfer [10]. From here, the immunotherapy refers to immune checkpoint blockade (ICB) therapy in this manuscript. ICB can block certain proteins that cancer cells use to escape from the immune system [11]. Thus, ICB therapy allows the immune system to more effectively target and attack cancer cells, while leaving healthy cells untouched. ICB has been studied across various cancer types, encompassing LC [12]. ICB therapy currently is one of the most promising treatments for LC and has proven effective in clinical trials when combined with other treatments such as chemotherapy and radiation [13].

Recent clinical trials have shown that ICB therapy can improve overall survival in patients with metastatic non‐small cell lung cancer (NSCLC) [14]. Although the results are encouraging, further research is needed to fully understand the safety and efficacy of this treatment. In addition, there is a need to identify biomarkers that can predict if a patient will respond to ICB therapy. With the development and wide application [15, 16] of single‐cell RNA sequencing (scRNA‐seq) technology, we can capture every cell's state in the tissue [17]. The tumor immune microenvironment (TIME) [18] contains a complex immune cell environment, including innate immune response cells such as natural killer (NK) cells, macrophages, and dendritic cells; and cells involved in adaptive immune responses, such as CD8^+^ and CD4^+^ T cells [19]. The heterogeneity within the TIME is intricately linked to the varied response rates observed among cancer patients undergoing ICB therapy [20]. The classification of the TIME facilitates a more profound comprehension of the involvement of immune cells in response to immunotherapeutic interventions.

Previous studies have shown that tumor‐specific T‐cell clones overexpress T‐cell killing and depletion‐related genes, while nontumor‐specific T cells do not. Therefore, exhausted T cells can be used as a representative for tumor‐specific T cells. Liu et al. [21] utilized clustering techniques to identify subtypes of exhausted T cells and determined that PD‐1 antibodies have the potential to reverse the tumor‐specific T‐cell differentiation toward an attenuated state within the tumor microenvironment in response to immunotherapy.

In this study, our objective was to explore the metabolic states of tumor‐infiltrating T cells in NSCLC and their association with ICB therapy outcomes. Through comprehensive analyses, we characterized the metabolic heterogeneity within T‐cell populations and discerned three discrete metabolic subtypes, each exhibiting a correlation with the prognosis of NSCLC patients subjected to ICB treatment. These metabolic subtypes were classified as response‐associated clusters (R) and nonresponse‐associated clusters (NR), delineating their metabolic disparities and specific functional characteristics. Notably, we observed a significant downregulation of the *HSPA1A* gene expression within the NR clusters across all three T‐cell subtypes.

Furthermore, by integrating T‐cell receptor (TCR) repertoire analysis with pseudotemporal analysis, we revealed the interconversion relationships among R and NR metabolic clusters within clonal populations of T cells. This suggests that the metabolic states of T cells may undergo dynamic changes through metabolic reprogramming. Additionally, utilizing intercellular communication analysis, we identified specific communication patterns within the R clusters, which could potentially activate and regulate signaling pathways, thus facilitating the progress of ICB therapy.

By revealing key insights into the metabolic dynamics of T cells following ICB therapy, our study contributes to a deeper understanding of immune responses in NSCLC patients undergoing treatment. Further investigation into the role of metabolic reprogramming and intercellular communication will aid in the development of personalized tumor immunotherapy strategies, facilitating the surmounting of treatment resistance and enhancement of patient outcomes.

## 2 Results

### 2.1 Classification of Metabolic Heterogeneity Within Immune Cells

Previously, Liu et al. [21] analyzed 47 tumor biopsy samples from 36 patients with NSCLC, covering 33 untreated, 9 immunotherapy‐responsive, and 5 immunotherapy‐unresponsive tumors, using scRNA‐seq and scTCR‐seq. Their study revealed that immunotherapy significantly increased the proportion of exhausted T‐cell precursor (Texp), which are defined as the unexhausted CD8^+^ T cells sharing TCRs with terminal exhausted T (Tex) cells, in responsive patients.

In our study, we selected 22 samples from Liu et al., which contains 17 samples from immunotherapy responders (8 before treatment and 9 after treatment) and 5 samples from immunotherapy nonresponders. And in these 22 samples, we obtained 77,367 high‐quality T cells, including 13 subtypes such as naive T cells (Tn), regulatory T cells (Treg), exhausted T cells (Tex), and nonexhausted T cells (Tnex).

**FIGURE 1:**
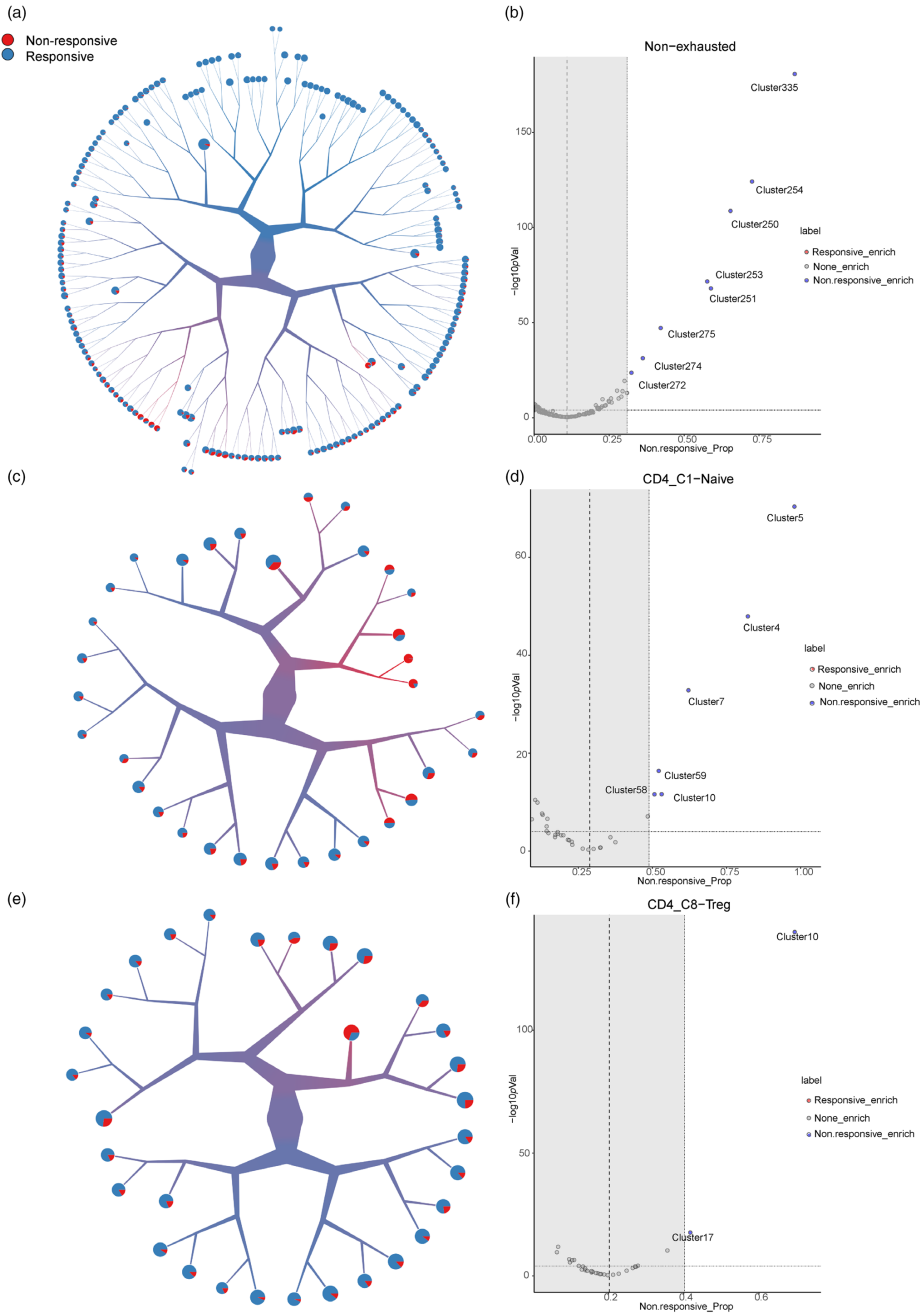
Metabolic clustering results of three T‐cell subtypes. (a) Visualization and clustering of TooManyCells tree structure of Tnex cells. Cells start from the initial root node and are divided according to similarities and differences in metabolic transcription profiles. The colors within the branches indicate the proportion of cells from either responding patients (blue) or nonresponding patients (red). The pie chart at the end of the branch shows the proportion of cells in this terminal cluster that respond to patients versus nonresponding patients. (b) The volcano map represents a deviation in the proportion of unresponsive patient cells between each cluster terminal and the original Tnex. The dashed line parallel to the *Y*‐axis is the original percentage of nonresponding patient cells in Tnex. A dashed line parallel to the *X*‐axis indicates a *p* value = 0.001, a one‐sided *z*‐test. Points represent each cluster terminal. (c) Same as in (a) but for Tn cells. (d) Same as in (b) but for Tn cells. (e) Same as in (a) but for Treg cells. (f) Same as in (b) but for Treg cells.

First, we evaluated the metabolic status of every T cell by extracting the expressions of 3294 metabolic genes, which were collected and defined by scMetabolism [22]. Since TooManyCells [23] algorithm is able to effectively, globally, and unbiasedly identify and visualize evolutionary cell clades based on scRNA‐seq data, we re‐clustered the cells of each T‐cell subtype utilizing TooManyCells algorithm based on the similarity of metabolic statuses. The clustering result of a T‐cell subtype is visualized as a tree‐like structure, with each leaf node being the most refined cluster. After clustering, as shown in Figure 1a–e, we used prognostic label to color the proportion of cells from responders and nonresponders in each leaf node. Upon scrutinizing the visualized clustering outcomes, we observed variations in the distribution of cells between responders and nonresponders across different clusters (Figure 1a–e, Figure S1a–g). To quantify the disparity in the proportion of cells between response and nonresponse patient groups within each metabolic cluster across every T‐cell subtype, we conducted a statistical test within these T‐cell subtypes.

For Tnex, in clusters 250, 251, 253, 254, and 335, the proportion of cells from nonresponders significantly deviated from the original proportions of nonresponder cells (Figure 1b). These results suggest that Tnex has metabolic heterogeneity and their metabolic status are linked to the prognosis. Therefore, we further explored Tnex in the subsequent analysis. According to the cellular proportion of responsive patients and nonresponsive patients in each metabolic cluster, the clusters in which the proportion of cells from nonresponsive patients are higher than 50% and have significant differences in statistical tests were classified as NR clusters, whereas the remaining clusters were designated as R clusters.

In addition to Tnex, we also found the metabolic heterogeneity in Tn and Treg (Figure 1c–f). Overall, subtypes of T cells which have prognosis‐related metabolic heterogeneity have been explored. And we then characterized the specific functions of these cells with prognosis‐related phenotypes.

### 2.2 Metabolic Heterogeneity in Response and Nonresponse‐Related Subpopulations

To investigate the metabolic differences between cells from R and NR clusters within each T‐cell subtype, we first performed differential gene expression analysis between R and NR metabolic clusters of Tnex using limma [24] package.

**FIGURE 2:**
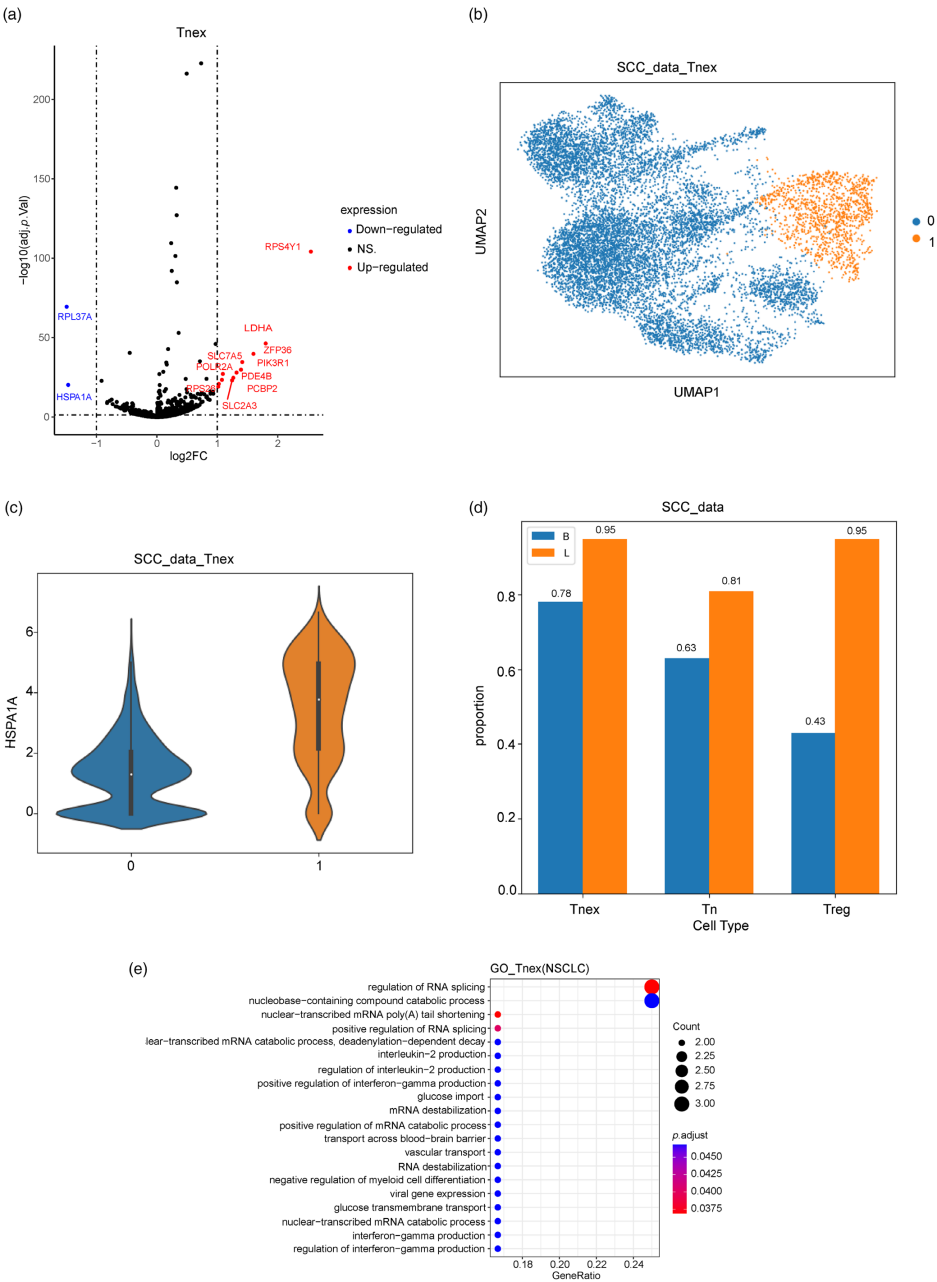
Metabolic heterogeneity of NR and R subgroups. (a) The differential gene volcano map shows the difference in gene expression between the NR and R subpopulations of Tnex cells. In this figure, red dots represent genes that are significantly upregulated in the NR subpopulation, and blue dots represent genes that are significantly downregulated. See Table S1 for details. (b) Clustering results of Tnex cells in SCC dataset based on Leiden algorithm. (c) The violin diagram shows the differential expression distribution of HSPA1A gene in different clusters in (b), and the *p* value was far less than 0.05. (d) According to the expression of HSPA1A in (c), the two subgroups in (b) were defined as NR (0) and R (1). For naive and Treg subtypes, we also recluster each subtype by Leiden algorithm and define R and NR according to the expression of HSPA1A. The bar chart shows the proportion of cells from responding patients before clustering of the three T‐cell subtypes and the proportion of cells from responding patients in the R subgroup after clustering. B is the proportion of cells before clustering and L is the proportion of cells from responding patients in the R subgroup after clustering (SCC). (e) GO (gene ontology) pathway enrichment analysis of significantly upregulated genes in the NR subpopulation of Tnex cells. The vertical axis is biological processes.

Among Tnex, the expressions of *PIK3R1*, *LDHA*, *PDE4B*, *PCBP2*, and other genes in NR cells are significantly higher than those in R cells (Figure 2a, Table S1). PIK3R1, also known as phosphoinositide 3‐kinase regulatory subunit 1, is an important regulatory subunit in the PI3K/AKT pathway, which is widely regarded as a key signaling pathway in tumor cells [25]. As one of the key enzymes involved in glycolysis, *LDHA* in T cells may affect the immune escape of the tumor cells [26]. For example, in melanoma, *LDHA*‐associated lactic acid accumulation inhibits tumor surveillance by T and NK cells [27]. Hence, NR cells are more likely to promote tumor growth and survival.

In addition to Tnex, we also performed differential gene expression analysis between R and NR clusters on Treg and naive T cells, respectively. Interestingly, we found *HSPA1A*, encoding a heat shock protein, is significantly downregulated in NR clusters within these three T‐cell subtypes. HSPA1A is a molecular chaperone that plays an important role in antigen presentation. It has been demonstrated to bind intracellular and extracellular antigens, which are then recognized by antigen‐presenting cells via CD91 or scavenger receptors, leading to endocytosis [28, 29]. Antigen‐specific T cells recognize the MHC molecular antigen ligand on the surface of antigen‐presenting cells or target cells and then introduce activation signals to T cells through CD3 molecules, which eventually lead to the activation of CD8^+^ T cells [30]. Using the previously published squamous cell carcinoma (SCC) dataset, we extracted Tnex cell subtypes, counted the proportion of cells from responding patients, and clustered Tnex cell subtypes using Leiden algorithm. We observed that Tnex cell subtypes in the SCC dataset were divided into two subgroups, and the expression of *HSPA1A* gene in these two subgroups was significantly different (Figure 2b,c). The two subpopulations were labeled R and NR based on the expression characteristics of the gene in the NSCLC dataset. Further statistics on the proportion of cells in R from responding patients showed a significant increase in the proportion after clustering compared with the proportion before nonclustering, which is consistent with the results of Tnex cell subtype analysis in previous NSCLC data (Figure 2d). The downregulation of *HSPA1A* suggests a shift toward a less activated and stress‐responsive phenotype, which could impact their differentiation, function, and survival.

To measure the distinct functions of R and NR cells, we conducted gene ontology (GO) enrichment analysis on differentially expressed genes in R and NR cells within each T‐cell subtype, respectively. In Tnex, the upregulated genes in NR cells are mainly concentrated in RNA splicing regulation and catabolism pathways of compounds containing nuclear bases, which implies the activities of NR cells of Tnex are suppressed (Figure 2e).

For naive T and Treg, the differences in metabolic genes between NR and R cells were also explored, and some related biological processes were identified based on the significantly upregulated genes in NR cells.

In naive T cells, the expression levels of metabolic genes such as *CH25H*, *UGP2*, *ABCC1*, and *SESN3* are significantly upregulated in NR cells compared with R cells (Figure S2a, Table S2). As CH25H (cholesterol 25‐hydroxylase) is a noncarrier gene involved in cholesterol and lipid metabolism, it plays an important role in lipid metabolism by synthesizing corepressors that block the processing of proteins binding to the sterol regulatory elements and ultimately lead to the suppression of gene transcription [31]. The catalytic enzyme UDP‐glucose pyrophosphorylase 2 (UGP2) drives the production of UDP‐glucose and plays a central role in carbohydrate synthesis and metabolism [32]. ABCC1 (ATP Binding Cassette Subfamily C Member 1) is a member of the superfamily of ATP‐binding cassette (ABC) transporters. The protein transports various molecules through the outer and inner membranes of the cell. Previous studies have shown that the expression level of *ABCC1* in T cells is related to drug resistance and therapeutic effect of tumor drugs [33, 34, 35]. The overexpression of *ABCC1* enhances the output of cGAMP and restricts the conduction of STING signal. cGAMP is an important secondary signaling molecule, which is generated by cyclic dinucleotide (cGAMP) catalyzed by cyclic adenylate synthetase (cGAS) and is an important molecule to initiate the STING signaling pathway [36, 37]. Overall, upregulated genes in NR‐native T cells are essential for the proper functioning of cells. Through the enrichment analysis of upregulated genes, we found that significantly upregulated genes in NR cells are mainly enriched in nucleotide metabolism‐related pathways (Figure S2c), which suggests that these cells have high‐energy demands related to cell proliferation and DNA synthesis. Considering that these biological processes are important for the maintenance of the genomic integrity of the cells, these naive T cells may be involved in initiating a primary immune response to a new antigen.

In Treg, the expression of metabolic genes such as *GSTP1*, *MGST1*, and *AKR1C2* are significantly upregulated in NR cells (Figure S2b, Table S3). Glutathione *S*‐transferase members GSTP1 and MGST1 may specifically alter enzyme activity in patients receiving platinum‐based chemotherapy, thus altering various toxicity [38], while *AKR1C2* is involved in the metabolism of steroid hormones. The upregulation of these genes suggests an increased need for cellular detoxification and altered hormonal signaling, which may affect the overall immune response in the TIME. We conducted GO enrichment analysis on these significantly upregulated genes, revealing their predominant involvement in the metabolism of prostaglandins, prostatic hormones and fatty acids. These pathways are related to regulation and control of the immune response, thus are important for the production of regulatory molecules and may be required for the immunosuppressive function of Tregs. Meanwhile, these significantly downregulated genes are mainly enriched in cytoplasmic translation, ATP metabolism, precursor metabolism, and energy production, suggesting that NR cells of Treg are more metabolically active (Figure S2d,e).

### 2.3 Improve Immunotherapy Efficacy Through Metabolic Reprogramming

Liu et al. also provided scTCR‐seq data paired with scRNA‐seq data for T cells. Based on the matching of chain A and chain B in these scTCR‐seq data, naive T, Tnex, and Treg cells were further divided into several different clone types, and the number of cells of each clone type was deduced. In naive T cells, Treg cells, as the number of cells in each clone of those two subtypes is small, we mainly analyzed the clones from Tnex cells.

In Tnex, clones with more than 50 cells were kept for downstream analysis. We found each clone contains both R and NR cells, suggesting that R and NR cells do not have specific progenitors. We thus suspected that the different metabolic status within a single T‐cell clone can be shifted by metabolic reprogramming. Therefore, we next inferred the state‐transition trajectory in these clones.

**FIGURE 3:**
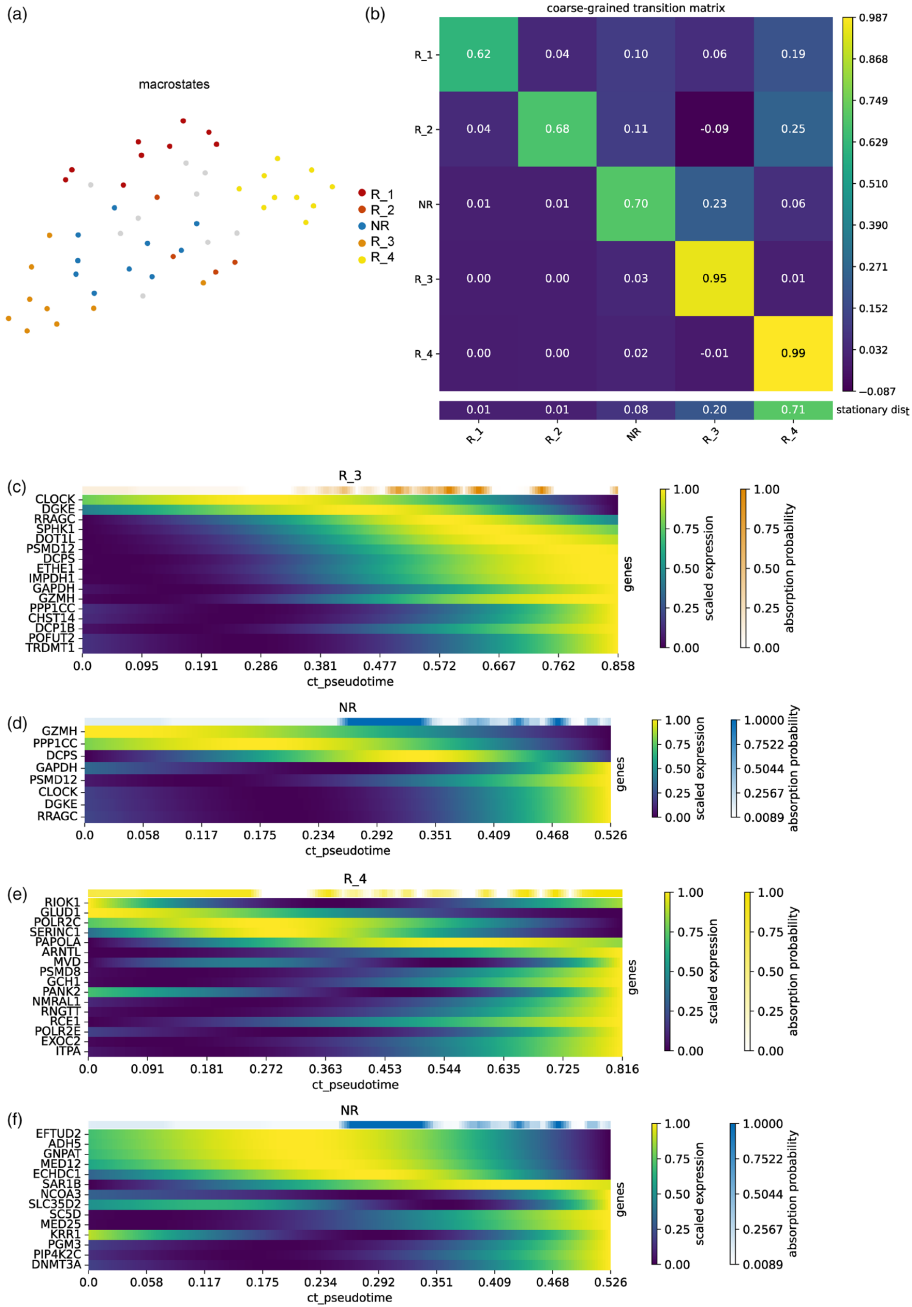
Metabolic changes of NR and R in the same clone of Tnex. (a) The distribution landscape of different macrostates in the same clonotype contains two subgroups NR and R in several macrostates. Each point represents a cell, and different colors represent different macroscopic states. (b) The probability of transfer between five different macroscopic states. (c) The expression trends of the top 20 genes (except ribosome‐related genes) whose expression values were most correlated with the fate probability of R_3 were sorted according to pseudotime peaks. (d) The expression trends of the top 20 genes (excluding ribosome‐related genes and nonconvergent genes) whose expression values were most correlated with R_3 fate probability in NR were sorted according to pseudotime peaks. (e) Same as in (c) but for R_4. (f) Same as in (c) but for NR.

CellRank can predict the pseudotime of every cell along a trajectory according to the similarity of gene expression profiles [39]. As the original single‐cell gene expression matrix is usually sparse and noisy, CellRank can combine similar single‐cell gene expression profiles into a macrostate to tackle this issue. We first constructed several macrostates to depict the metabolic landscape in each clone type. The beginning and end macrostates along the trajectory were defined based on the inferred distribution of pseudotime and the transfer probability between pairwise macrostates. The macrostate cluster with a higher probability of self‐transition and lower probability of transition to other macrostates is defined as the end of the trajectory, while the macrostate cluster with a lower probability of self‐transition and higher probability of transition to other macrostates is defined as the beginning of the trajectory (Figure 3a,b). In two Tnex clones, we observed the cross‐transformation relationship between R cells and NR cells and identified the specific regulatory factors of cell fate (Figure 3c–f).

### 2.4 The Potential Factors That can Drive Metabolic Reprogramming

In the tumor microenvironment, besides the communication between tumor cells and immune cells, the autocrine signaling among T cells also plays an important role in tumor development [40]. In this study, differentially expressed genes between R and NR T cells may be caused by the differences in communication patterns of these two groups of T cells. In order to identify potential communication between T cells of different phenotypes, genes significantly upregulated in NR cells of naive T, Tnex, and Treg were used as gene sets of interest to obtain potential ligand–target gene interaction information based on ligand–receptor interaction network and gene regulatory network.

The foundation of cellular heterogeneity in tissues lies in the diversity of cell transcriptional states, and the specificity of these states is established and sustained by the gene regulatory network dominated by transcription factors (TFs) [41]. Therefore, in order to explore the biological significance behind the heterogeneity of R and NR cells, potential targets of each TF were identified based on gene coexpression. We used SCENIC [42] to identify TFs expressed in these three T‐cell types and to infer their regulatory networks in cell fate decisions (Figures S3–S5). In Tnex, TFs regulating expression of NR cells‐related genes showed low activities of *IRF5*, *EOMES*, and *ETV1* (Figure S5). The PI3K‐AKT signaling pathway has been reported to inhibit IRF5 transcriptional activity by phosphorylating *IRF5* [43]. Our previous analysis of gene differential expression of NR and R cells in nonexhausted T cells has found that *PIK3R1* is upregulated in NR cells, which may activate the PI3K‐AKT signaling pathway.

We observed the existence of *STAT3* and CSK in the top 10 receptors of naive T, Tnex and Treg cell subtypes with the highest activity scores (Figures S6–S8). Transcriptionally, *STAT3* regulates the expression of genes associated with cell proliferation, anti‐apoptosis, pro‐survival, angiogenesis, metastasis, and immune escape [44]. As an endogenous inhibitor of SFK, CSK's known regulation of apoptosis and survival function depends on the inhibition of SFK. CSK's inhibition of SFK has a pro‐apoptotic effect, which is mediated by the cascade inhibition of cell signals controlled by SFK, such as MAPK/ERK, STAT3, and PI3K/AKT signaling pathway [45].

**FIGURE 4:**
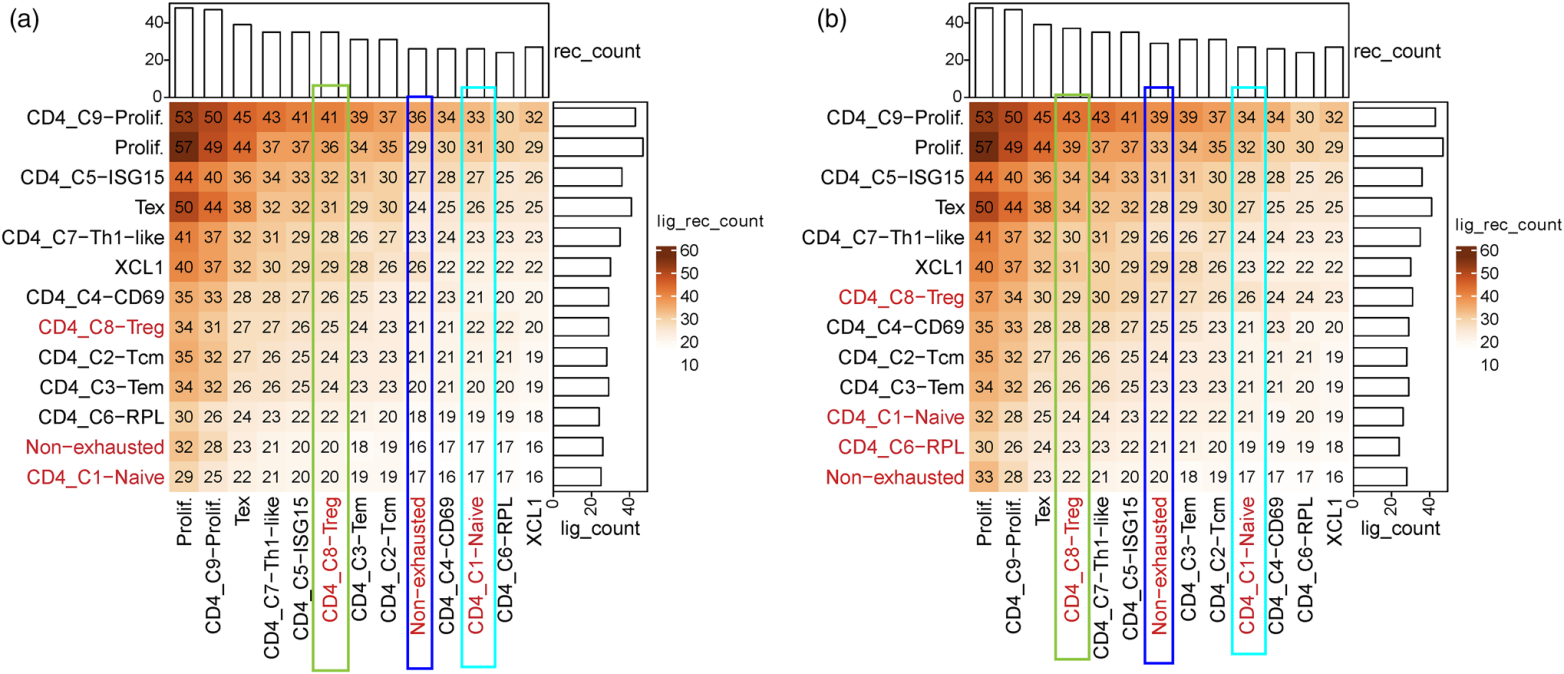
Communication between T‐cell subtypes. (a) Heat map of communication between Tnex cell NR subsets and other T‐cell subtypes. The numbers in the matrix are the number of ligand–receptor pairs per cell type, colored relative to the maximum count. Rows represent cells expressing ligands and columns represent cells expressing receptors. (b) Same as in (a) but for R subsets.

We previously elucidated the metabolic reprogramming of these three T‐cell subtypes through intracellular gene expression. In order to further explore which extracellular signals is linked to the intracellular regulation, we used NATMI [46], a tool to help users explore cell‐to‐cell communication using scRNA‐seq or other omics expression data based on the ligand–receptor pairs in connectomeDB2020 database, to identify the intercellular communication among different T‐cell subtypes through ligand–receptor interactions. We first used NATMI to identify ligand–receptor teams between NR and R cells in the Tnex subtype and other T‐cell subtypes. NATMI analysis showed that R cells had significant characteristics in their interactions with other cell subtypes. Specifically, we found that R cells communicated specifically with other T‐cell subtypes compared with NR cells (Figure 4a,b). This specificity is mainly manifested in *HLA‐E–KLRD1*, *HLA‐B–KLRD1*, *VCAM1–ITGB1*, and *LGALS1*–*ITGB1* (Table S4). The interaction between these ligands and receptors may lead to the activation and regulation of signal transduction pathways, thereby affecting the effectiveness of tumor immune response.

## 3 Discussion

In this study, we investigated the metabolic heterogeneity of tumor‐infiltrating T cells in patients with NSCLC who received ICB therapy. By clustering T‐cell subtypes based on their metabolic gene expressions, we identified three T‐cell subtypes exhibiting metabolic heterogeneity. Based on the metabolic heterogeneity, we classified cells of these subtypes into R clusters and NR clusters. Differential gene expression analysis revealed significant upregulation of certain genes, namely *LDHA*, *ZFP36*, *PIK3R1*, *CH25H*, and *ABCC1*, within the NR clusters of these three T‐cell subtypes. Notably, we consistently observed downregulation of the *HSPA1A* gene within the NR clusters across all three T‐cell subtypes. Through the trajectory analysis, we uncovered a potential role of metabolic reprogramming in altering the ICB therapy efficacy, which suggested that the differential metabolic states of T cells may contribute to the variable response to ICB therapy in NSCLC patients. Additionally, our exploration into intercellular communication patterns among T cells revealed specific communication modes within the R clusters, and identified interactions between receptors such as *HLA‐E*, *KLRD1*, *VCAM1*, and *ITGB1*, which may activate and regulate signaling pathways such as MAPK, NF‐kB, and mTOR, thus influencing the functions of T cells.

The findings of our study have important implications for understanding the mechanisms underlying the response to ICB therapy in NSCLC patients. The identification of distinct metabolic subtypes within tumor‐infiltrating T cells provides valuable insights into the heterogeneity of immune cell functionality. The consistent downregulation of *HSPA1A* across the NR clusters suggests its potential role as a biomarker and drug target for ICB therapy resistance. Furthermore, the interconversion between metabolic clusters and the involvement of metabolic reprogramming highlights the plasticity of T‐cell metabolism and its impact on treatment outcomes.

The intercellular communication patterns within the R clusters further supports the notion that autocrine signaling plays a crucial role in modulating not only the functions but also the metabolic status of T cells. The identified receptor may serve as a potential target for therapeutic interventions aimed at enhancing the effectiveness of ICB therapy. However, it is important to acknowledge the limitations of our study. The single‐cell analysis focused specifically on T cells within the tumor microenvironment, and additional investigations considering other cellular populations in the tumor microenvironment and their interplay are warranted. Furthermore, the functional consequences of the identified gene expression changes and intercellular communication patterns require further experimental validation to establish their direct role in immune response modulation.

In conclusion, our study provides novel insights into the metabolic heterogeneity of tumor‐infiltrating T cells and their association with the response to ICB therapy in NSCLC patients. The identification of specific metabolic subtypes, altered gene expression patterns, and communication networks form the groundwork for future investigations geared toward formulating personalized treatment strategies that target metabolic reprogramming and intercellular communication, with the objective of overcoming ICB therapy resistance in NSCLC. Further studies can apply spatial transcriptomics sequencing to reveal the spatial features that are associated with the response to ICB therapy.

## 4 Methods

### 4.1 Single‐Cell Data Processing and Clustering Visualization of Different Cell Types

Using scRNA‐seq data from Liu et al., we normalized the gene expression level to 1e6 and performed logarithmic conversion. We then screened 11 patients who had received immunotherapy and extracted 22 tumor samples from these 11 patients, both pre‐ and post‐treatment. From these samples, we extracted the RNA‐Seq data of 13 T‐cell subtypes and labeled each cell according to the clinical information of the patient to which it belonged. We used 3294 metabolic genes extracted from the R package scMetabolism [22] designed by Wu et al. The metabolic gene set was derived from a collection of 85 KEGG metabolic pathways and 82 REACTOME metabolic pathways. A total of 3241 metabolic genes were retrieved from T‐cell sequencing data, and the metabolic gene expression matrix was used to define the metabolic state of T cells.

We used TooManyCells (version 2.2.0.0, Docker) [23]. Each T‐cell type was clustered according to the metabolic gene expression matrix, where min‐size was set to “2” and other Settings were default, and each leaf node was colored according to the patient's immunoefficacy‐responsive patient versus nonresponsive patient. The proportion of the number of unresponsive patient cells in the total number of cells before clustering of each T‐cell subtype was calculated. Following clustering, we calculated the ratio of unresponsive patient cells to the total number of cells within each leaf node. The proportion test was conducted by the prop.test function in R language.

### 4.2 Differential Expression Analysis of Different Cell Types

For the analysis of gene differential expression [47] at the single‐cell level, we used the limma package [24] based on R language (version 3.50.3). Input the metabolic gene expression matrix along with the corresponding cell labels (R/NR), then perform intergroup comparisons using the grouping matrix generated by the “model.matrix” function and the “makeContrasts” function. The output coef parameter of the result is set to 1, and the adjustment method based on *p* value is Benjamini and Hochberg (BH). According to the adjusted *p* value and logFC, genes with adj.*p* < 0.05, logFC >1 were labeled as upregulated, while those with adj.*p* < 0.05, logFC <1 were labeled as downregulated.

### 4.3 scTCR‐seq Data Processing and Pseu HSPA1Ado Time Series Analysis

Based on the scTCR‐seq data provided by Liu et al., according to the matching between chain A and chain B, different clone types were divided for each T‐cell subtype. The number of each clone type was deduced according to whether chain A of each cell was the same. The clone type with more than 50 cells and containing both R and NR subgroups of cells was selected. We used the CellRank package [39] in python to infer pseudotime differentiation based on the expression similarity of metabolic genes in the absence of RNA rate information. The kernel was selected to calculate the probability of directed transition based on KNN diagram and CytoTRACE scores, and the estimator selected GPCCA.

### 4.4 Receptor Activity Analysis

NicheNet is a computational approach used to predict the impact of ligands on predefined gene sets. Initially, we applied NicheNet to evaluate the ligand activity on the upregulated differential metabolic genes that showed a logFC greater than 1 and an adjusted *p* value lower than 0.05, specifically within the NR‐E metabolic state. Next, we selected ligands with positive values and determined their activities on receptors. We then determined the activity of each receptor by referencing the ligand–receptor datasets from OmniPath.

### 4.5 TF Analysis

To assess the level of regulation by TFs, we utilized the pySCENIC (version 0.12.0) pipeline, which incorporates single‐cell regulatory network inference and clustering. The identification of differentially regulated gene sets was performed using the FindMarkers function from the Seurat package. Specifically, we defined the criteria for upregulated regulons associated with the NR metabolic state as having an average log2FC greater than 0.01 and an adjusted *p* value lower than 0.01.

### 4.6 Analysis of Intercellular Communication

Cellular interactions play important roles in many biological processes [15, 48]. To infer cellular interactions, we used python's toolkit NATMI, specifically the Docker image. Based on the ligand–receptor pairs in connectomeDB2020 data, a total of 1413 duplicate genes were screened from the input T‐cell sequencing data as the NATMI input data. In the output results, we considered that ligands and receptors with a detection rate of less than 20% in a given cell type were not expressed in that particular cell type, and ligands and receptors with a detection rate of more than 0.2 at the same time were retained.

### 4.7 Material Availability

This study did not generate new unique reagents.

## Author Contributions

Rui Hou, Liangyu Zhang, and Ziheng Wu conceived and designed this study. Hongzhe Guo and Peiwen Liu wrote the manuscript. Hongzhe Guo performed the data analysis with help from Hu Tang and Yue Gong. Bin Hu revised the manuscript. All other authors reviewed and approved the final version of the manuscript.

## Conflicts of Interest

Rui Hou and Yue Gong are employed by Genies Beijing Co., Ltd. All other authors declare no competing interests.

## Supporting information


Appendix S1.

## Data Availability

All datasets in this study, including RNA‐Seq and TCR‐seq data, were downloaded from public datasets. The NSCLC dataset and SCC dataset from Zhang et al. are available in GEO with the login code GSE179994 and GSE123813.
