# Supplementary figures and images for: Exploring the Role of T‐Cell Metabolism in Modulating Immunotherapy Efficacy for Non‐Small Cell Lung Cancer Based on Clustering
Source: J Clin Lab Anal. 2025 Apr 17;39(13):e25020. doi: 10.1002/jcla.25020 (PMC12217653; doi:10.1002/jcla.25020)

a

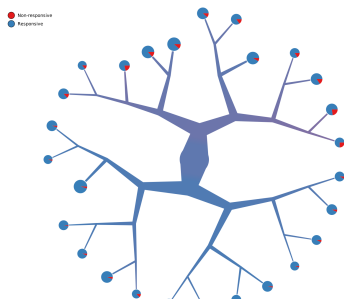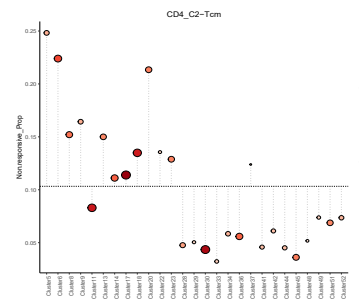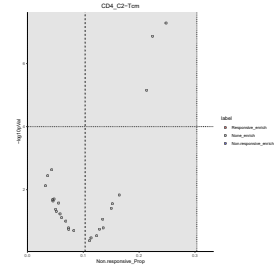

b

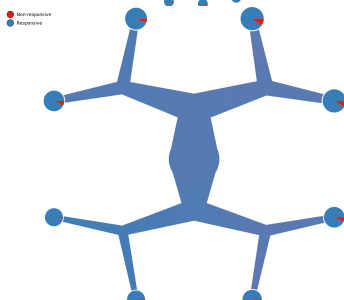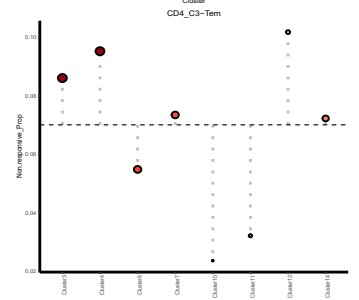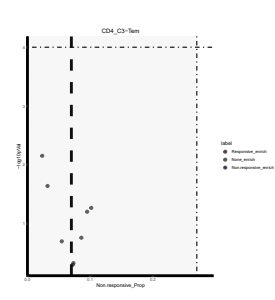

c

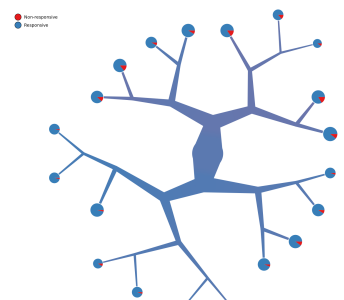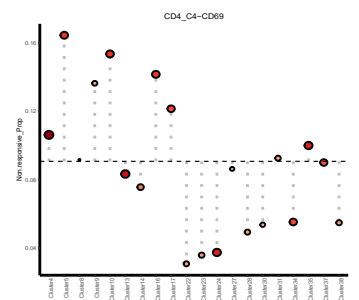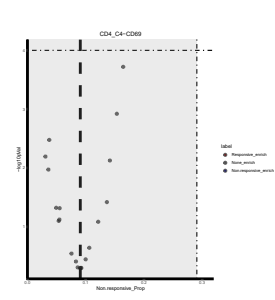

d

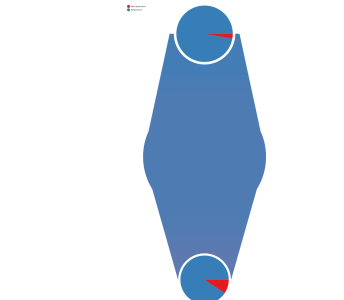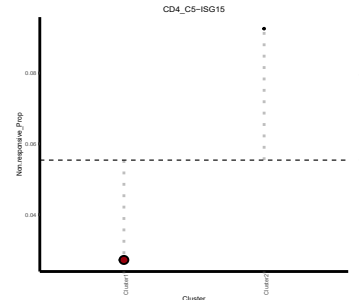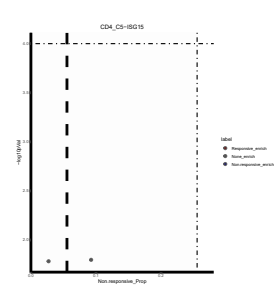

e

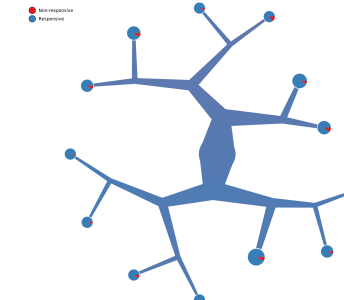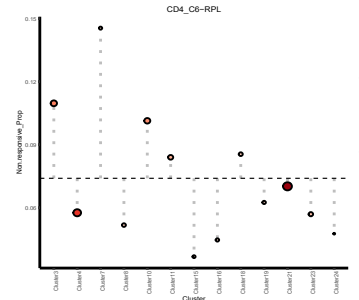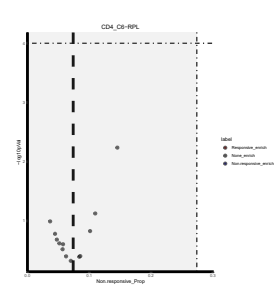

f

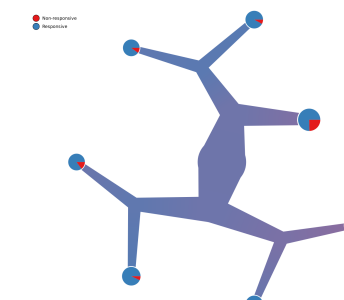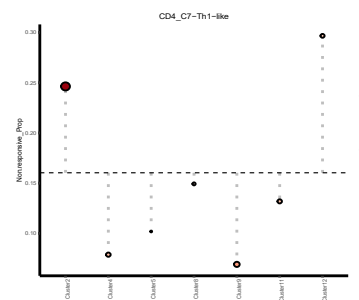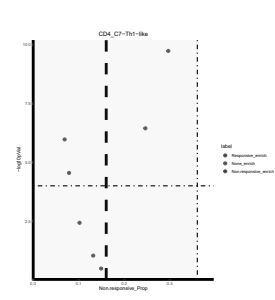

g

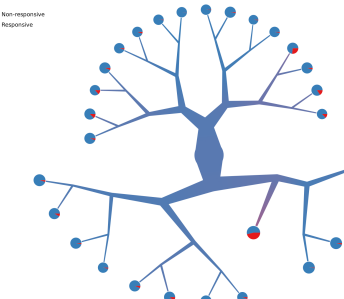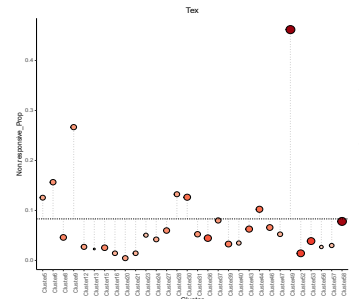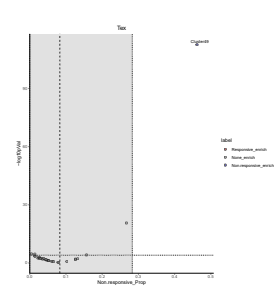

Supplement: Supplementary file 1 — Appendix S1. [file JCLA-39-e25020-s001.zip › supplementary/supplementary Fig1.pdf]

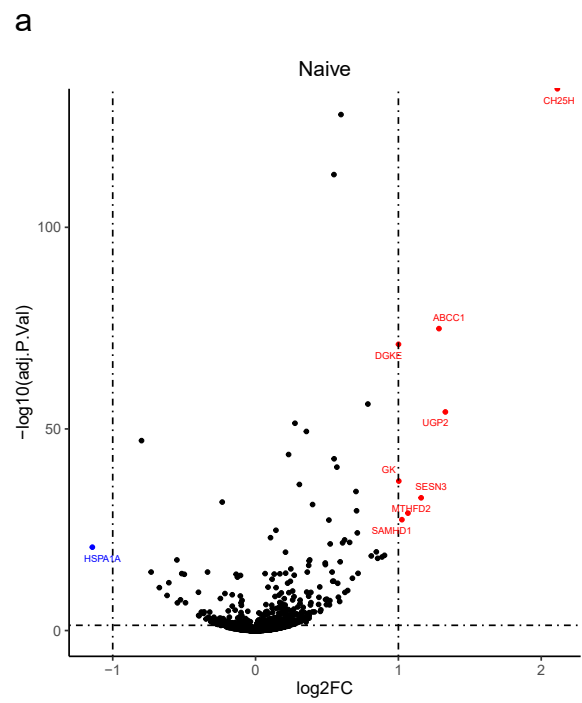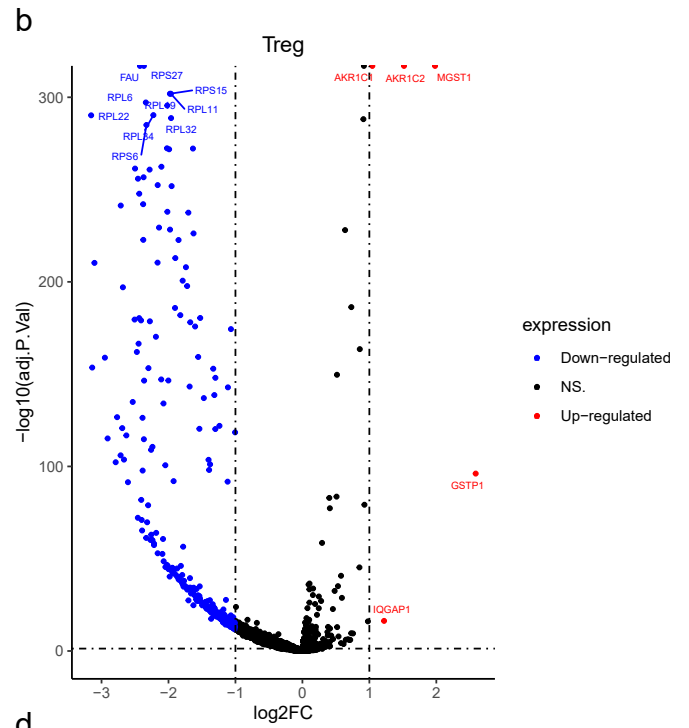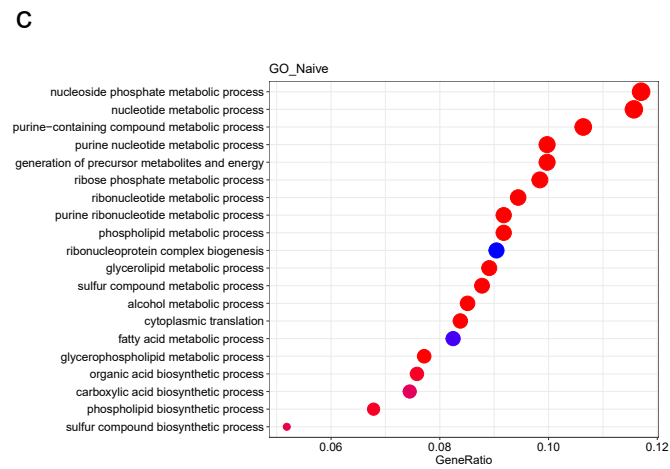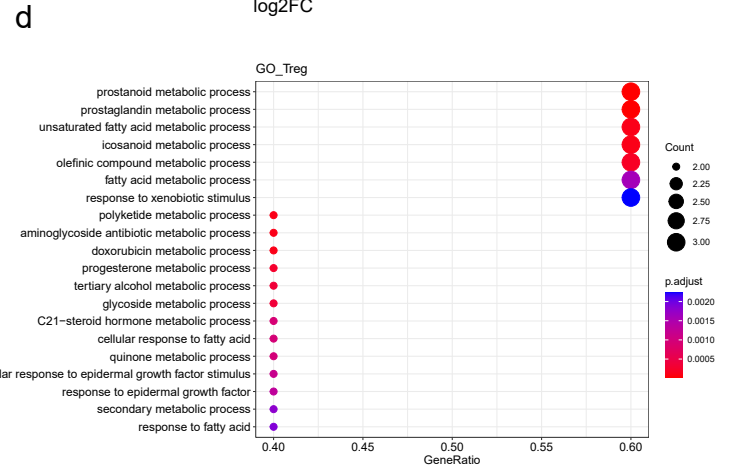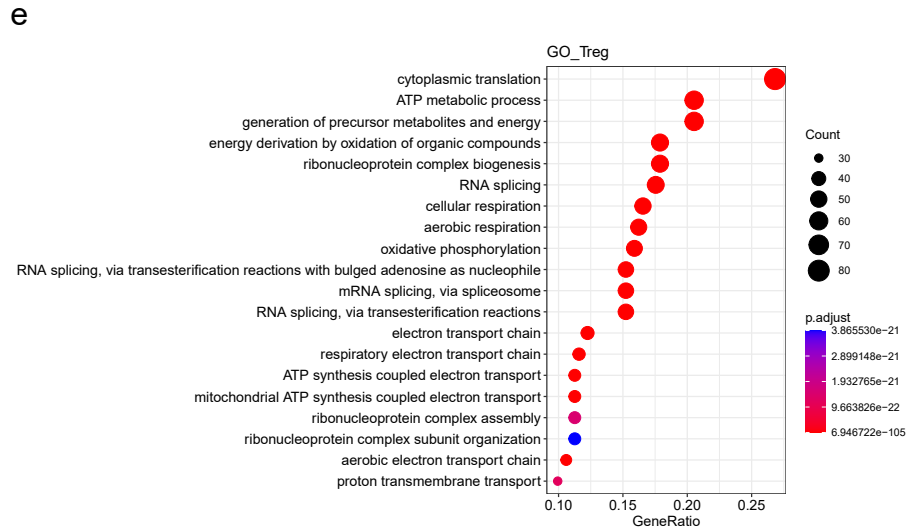

Supplement: Supplementary file 1 — Appendix S1. [file JCLA-39-e25020-s001.zip › supplementary/supplementary Fig2.pdf]

NR

R

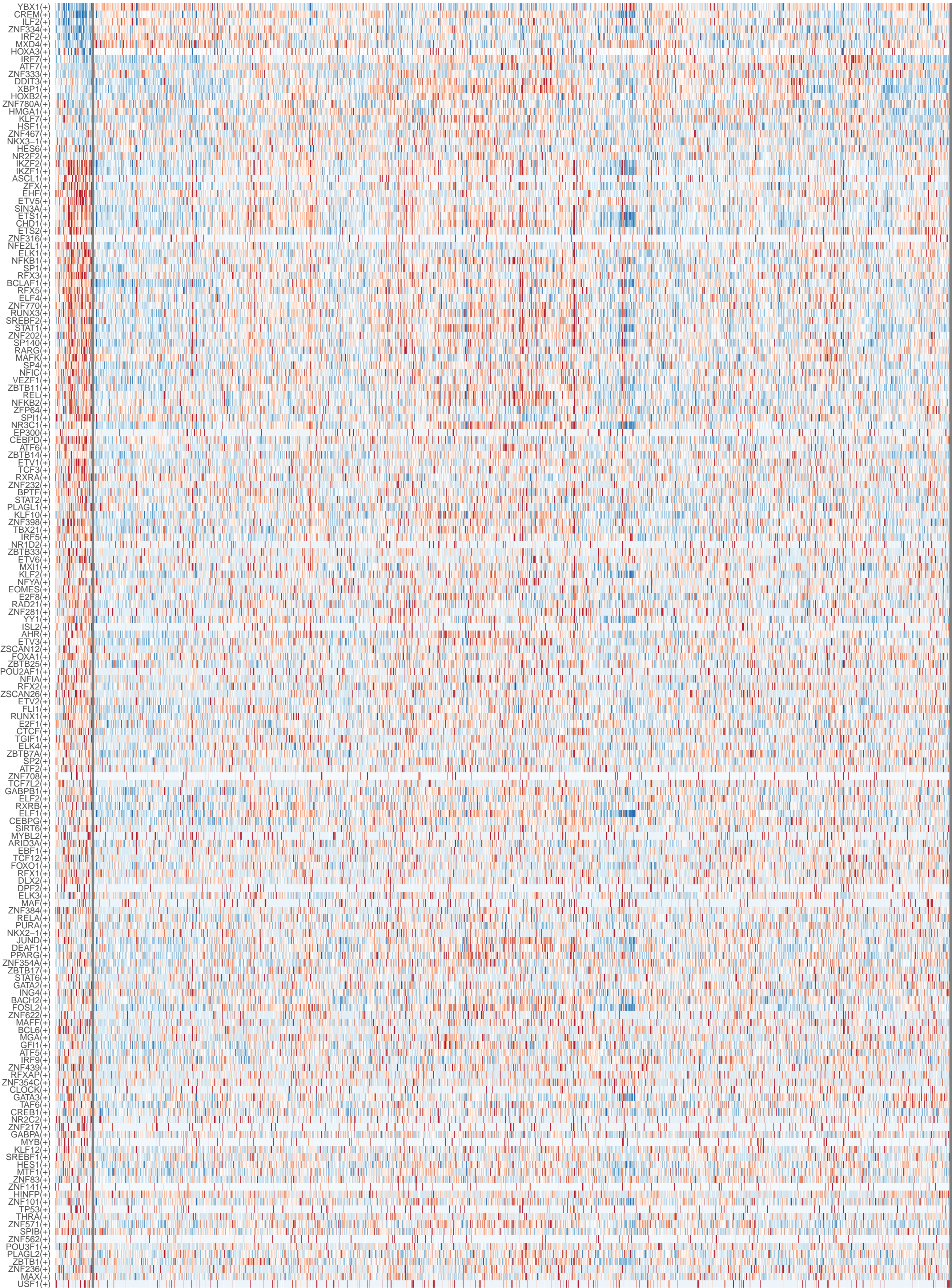

Identity

NR

R

Expression

2

1

0

-1

-2

Supplement: Supplementary file 1 — Appendix S1. [file JCLA-39-e25020-s001.zip › supplementary/supplementary Fig3.pdf]

NR

R

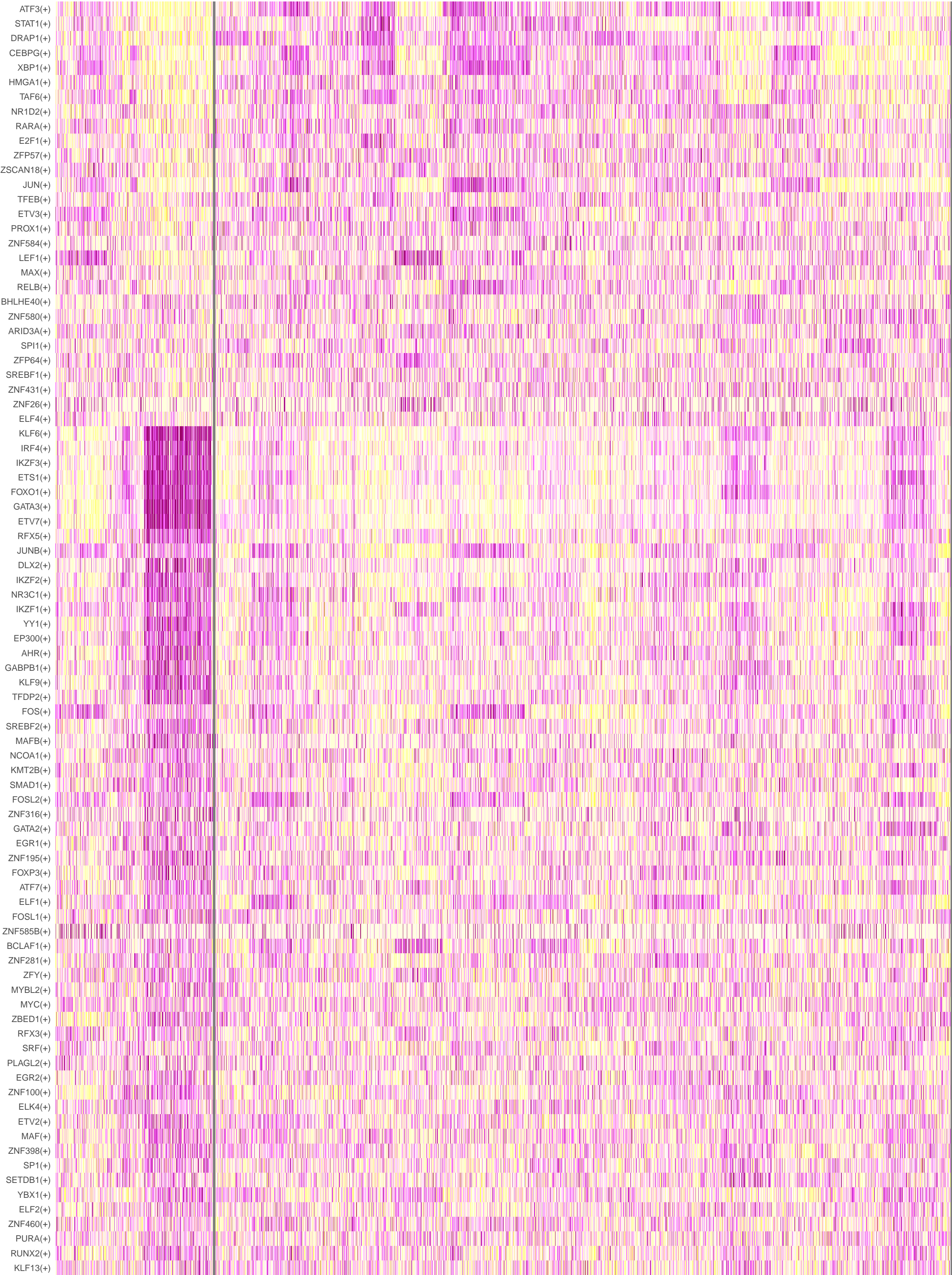

Identity

- NR
- R

Expression

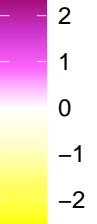

Supplement: Supplementary file 1 — Appendix S1. [file JCLA-39-e25020-s001.zip › supplementary/supplementary Fig4.pdf]

NR

R

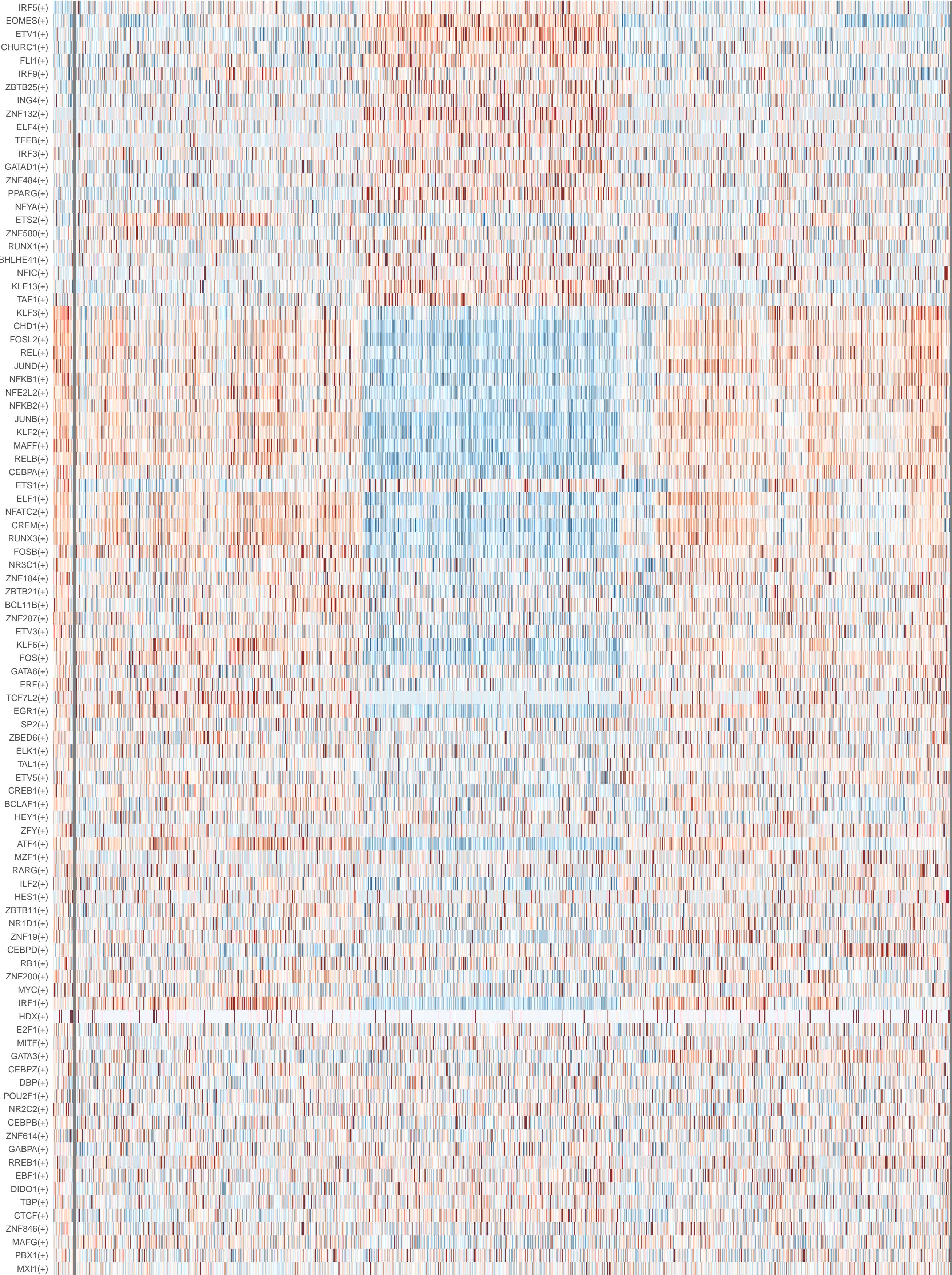

Identity

- NR
- R

Expression

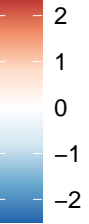

Supplement: Supplementary file 1 — Appendix S1. [file JCLA-39-e25020-s001.zip › supplementary/supplementary Fig5.pdf]

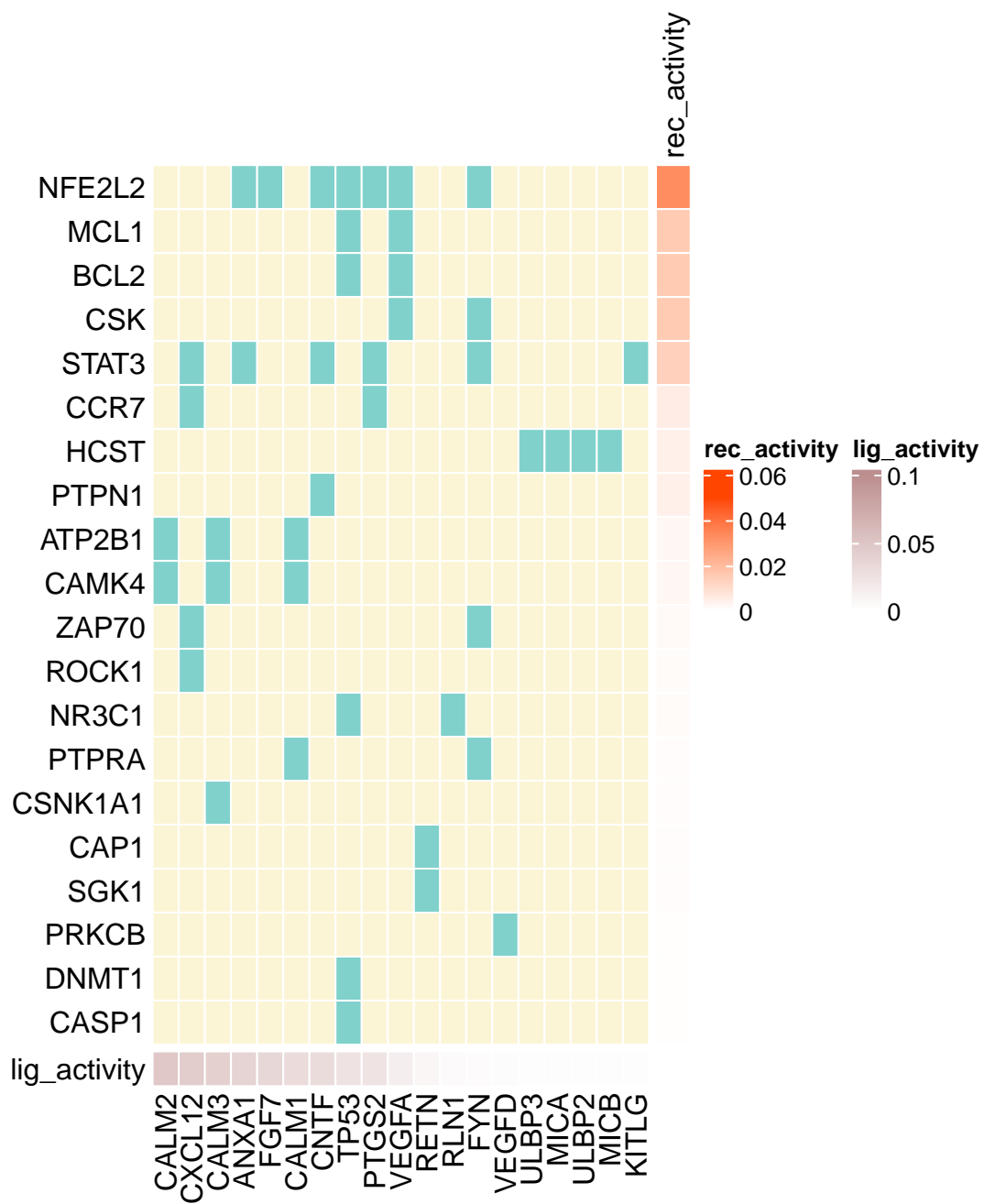

Supplement: Supplementary file 1 — Appendix S1. [file JCLA-39-e25020-s001.zip › supplementary/supplementary Fig6.pdf]

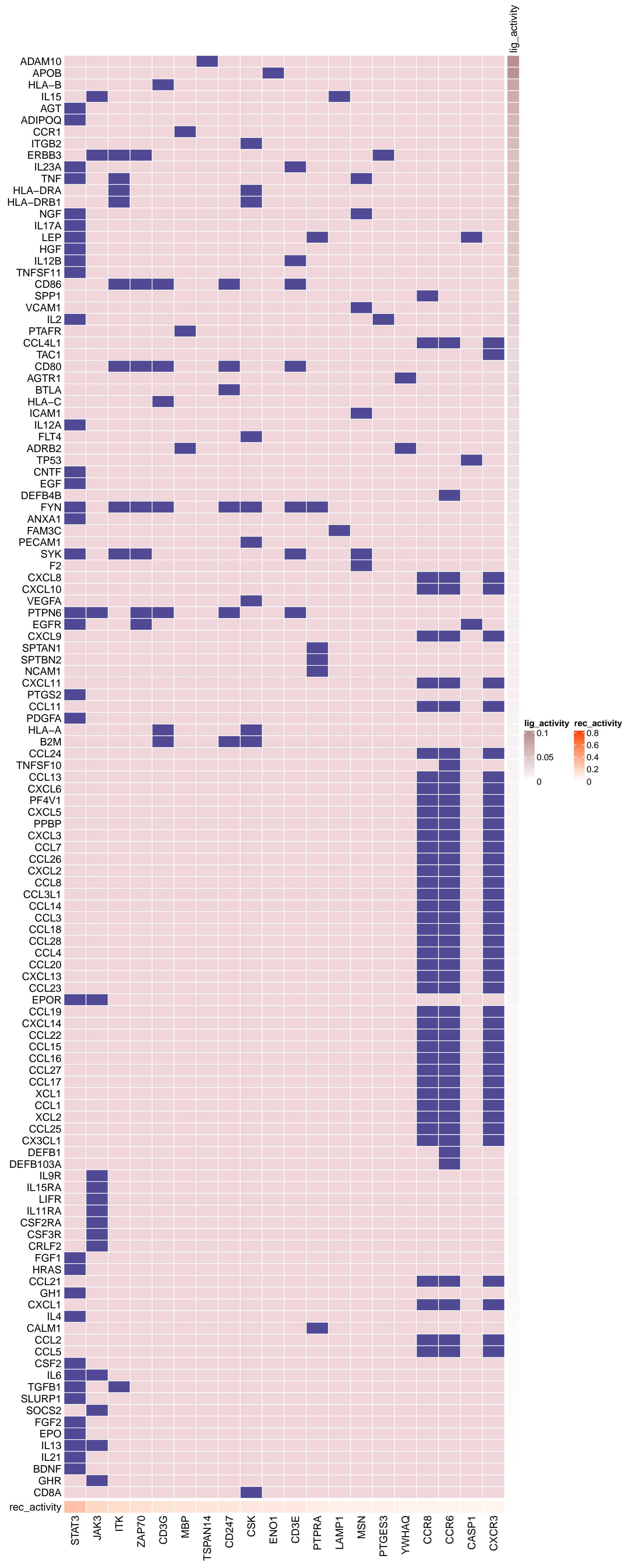

Supplement: Supplementary file 1 — Appendix S1. [file JCLA-39-e25020-s001.zip › supplementary/supplementary Fig8.pdf]
