# Supplementary material for: Exploring the Role of T‐Cell Metabolism in Modulating Immunotherapy Efficacy for Non‐Small Cell Lung Cancer Based on Clustering
Source: J Clin Lab Anal. 2025 Apr 17;39(13):e25020. doi: 10.1002/jcla.25020 (PMC12217653; doi:10.1002/jcla.25020)
Supplement: Supplementary file 1 — Appendix S1. [file supplementary Fig7.pdf › supplementary/supplementary Fig7.pdf]

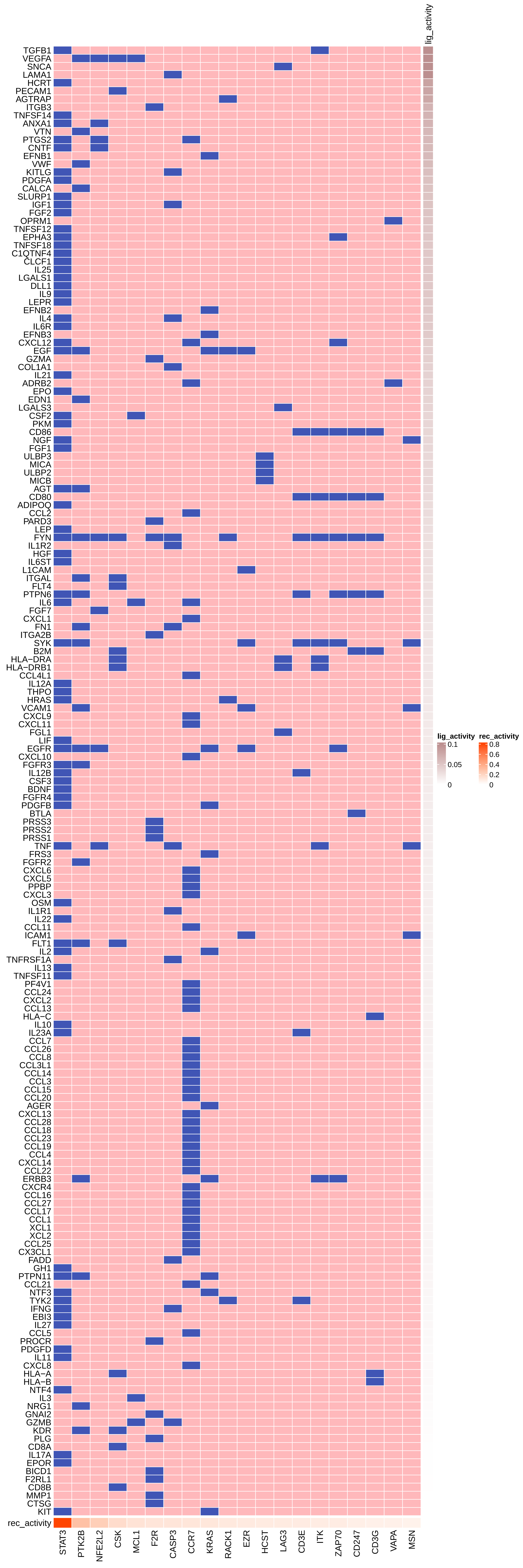

lig_activity
rec_activity
STAT3
PTK2B
NFE2L2
CSK
MCL1
F2R
CASP3
CCR7
KRAS
RACK1
EZR
HCST
LAG3
CD3E
ITK
ZAP70
CD247
CD3G
VAPA
MSN
0.1
0.05
0
0.8
0.6
0.4
0.2
0
